# BMI1 Drives Steroidogenesis Through Epigenetically Repressing the p38 MAPK Pathway

**DOI:** 10.3389/fcell.2021.665089

**Published:** 2021-04-13

**Authors:** Jun Yu, Yibo Wu, Hong Li, Hui Zhou, Cong Shen, Tingting Gao, Meng Lin, Xiuliang Dai, Jian Ou, Meiling Liu, Xiaoyan Huang, Bo Zheng, Fei Sun

**Affiliations:** ^1^Institute of Reproductive Medicine, School of Medicine, Nantong University, Nantong, China; ^2^Human Reproductive and Genetic Center, Affiliated Hospital of Jiangnan University, Wuxi, China; ^3^State Key Laboratory of Reproductive Medicine, Center for Reproduction and Genetics, Suzhou Municipal Hospital, The Affiliated Suzhou Hospital of Nanjing Medical University, Gusu School, Nanjing Medical University, Suzhou, China; ^4^Center of Clinical Reproductive Medicine, The Affiliated Changzhou Maternity and Child Health Care Hospital of Nanjing Medical University, Changzhou, China; ^5^State Key Laboratory of Reproductive Medicine, Department of Histology and Embryology, Nanjing Medical University, Nanjing, China; ^6^National Health Commission Key Laboratory of Male Reproductive Health, National Research Institute for Family Planning, Beijing, China

**Keywords:** testosterone, *Bmi1*, p38 mitogen-activated protein kinase (MAPK) signaling, epigenetic mechanism, steroidogenesis

## Abstract

Testosterone biosynthesis progressively decreases in aging males primarily as a result of functional changes to Leydig cells. Despite this, the mechanisms underlying steroidogenesis remain largely unclear. Using gene knock-out approaches, we and others have recently identified *Bmi1* as an anti-aging gene. Herein, we investigate the role of BMI1 in steroidogenesis using mouse MLTC-1 and primary Leydig cells. We show that BMI1 can positively regulate testosterone production. Mechanistically, in addition to its known role in antioxidant activity, we also report that p38 mitogen-activated protein kinase (MAPK) signaling is activated, and testosterone levels reduced, in BMI1-deficient cells; however, the silencing of the p38 MAPK pathway restores testosterone production. Furthermore, we reveal that BMI1 directly binds to the promoter region of *Map3k3*, an upstream activator of p38, thereby modulating its chromatin status and repressing its expression. Consequently, this results in the inhibition of the p38 MAPK pathway and the promotion of steroidogenesis. Our study uncovered a novel epigenetic mechanism in steroidogenesis involving BMI1-mediated gene silencing and provides potential therapeutic targets for the treatment of hypogonadism.

## Introduction

Reduced serum testosterone, known as hypogonadism, is a clinical condition in aging males resulting from reduced androgen production (Basaria, 2014). Hypogonadism is associated with several metabolic symptoms, such as the loss of muscle mass and bone mineral density, as well as with sexual dysfunction, mood changes, and reduced quality of life (Wu et al., 2010). Based on serum testosterone levels, approximately 20%, 30%, and 50% of men aged 60, 70, and 80 are defined as biochemically hypogonadal, respectively (Salonia et al., 2019). Although exogenous testosterone administration can reverse several hypogonadism-associated symptoms (Mayor, 2016; Snyder et al., 2016), emerging evidence has revealed that it may also increase the risk of cardiovascular disease and cancer, and also impede spermatogenesis (Kolettis et al., 2015; Nguyen et al., 2015; Budoff et al., 2017; Sargis and Davis, 2018). Studies focusing on the mechanisms of testosterone formation can help identify potential targets for use in the treatment of hypogonadism.

In males, testosterone biosynthesis occurs mainly in testicular Leydig cells through the regulation of the hypothalamus–pituitary–gonadal axis (HPG) (Nargund, 2015). Luteinizing hormone (LH) binds to the LH receptor (LHR) and stimulates the production of intercellular cAMP in a G protein-dependent manner. Subsequently, cAMP facilitates cholesterol translocation into the inner mitochondrial membrane. This is aided by steroidogenic acute regulatory protein (StAR), and represents the first rate-limiting step in steroid synthesis. In the mitochondria, cholesterol is converted to pregnenolone by the activity of the cytochrome P450 enzyme CYP11A1. Pregnenolone is subsequently metabolized to testosterone in the smooth endoplasmic reticulum through enzymatic transformation (Miller and Auchus, 2011; Miller, 2017; Zirkin and Papadopoulos, 2018). In both aging humans and rodents, serum testosterone levels diminish as a result of the reduced capacity of Leydig cells to produce testosterone (Wang et al., 2017). Although the mechanism underlying these age-related defects remains uncertain, there is evidence that this is mediated, at least in part, by reactive oxygen species (ROS)-induced oxidative damage to the Leydig cells (Beattie et al., 2015; Wang et al., 2017). For instance, it is well established that age-related oxidative stress inhibits steroidogenesis in adrenal and Leydig cells through the activation of p38 mitogen-activated protein kinase (MAPK), which further transcriptionally represses *STAR* gene expression (Abidi et al., 2008a, b; Zaidi et al., 2014).

B lymphoma Mo-MLV insertion region 1 (BMI1) is a member of the polycomb group (PcG) of epigenetic silencers first identified as an oncogene and subsequently revealed to be essential for stem cell maintenance (Haupt et al., 1991; van Lohuizen et al., 1991; Park et al., 2004). Studies have shown that *Bmi1* gene knock-out (KO) mice display premature aging phenotypes, including severe defects in hematopoietic cell self-renewal, excessive ROS production, and a shortened lifespan (van der Lugt et al., 1994; Molofsky et al., 2003; Park et al., 2003; Liu et al., 2009). More recently, we reported that mice deficient for BMI1 show reduced levels of serum testosterone and steroidogenic enzymes, as well as increased ROS production, leading finally to impaired spermatogenesis in the testes (Dai et al., 2018). We also found that the inhibition of BMI1 significantly reduces testosterone production in mouse MLTC-1 cells and primary Leydig cells, whereas treatment with the antioxidant N-acetylcysteine (NAC) can partially restore steroidogenesis (Gao et al., 2020). The oxidative stress-induced activation of p38 MAPK inhibits steroidogenesis suggests that BMI1 regulates steroidogenesis *via* the ROS-p38 MAPK pathway. In this study, we found that BMI1 deficiency indeed leads to the activation of the p38 MAPK pathway both *in vitro* and *in vivo*. We further found that the activation of the p38 MAPK pathway in BMI1-deficient cells occurs as a direct result of the epigenetic regulation of mitogen-activated protein kinase kinase kinase 3 (*Map3k3*), an upstream regulator of p38 MAPK, and that this activation was independent of ROS production. These results strongly suggested that BMI1 is a crucial epigenetic mediator of hypogonadism.

## Materials and Methods

### Mice

Mice were bred and maintained in a temperature- and humidity-controlled room in the Experimental Animal Center of Nanjing Medical University. Mice heterozygous for *Bmi1* (C57BL/6J background) were mated to generate wild-type (WT) and *Bmi1* KO mice. The mice were genotyped using PCR as previously described (Dai et al., 2018). The animal use protocol was reviewed and approved by the Ethics Committee of Nanjing Medical University.

### Cell Culture and Reagents

The mouse MLTC-1 Leydig cell line was purchased from ATCC (Manassas, VA, United States). These cells can synthesize testosterone and retain the full set of steroidogenic enzymes (Mendoza-Villarroel et al., 2014). The cells were maintained in DMEM medium supplemented with 10% fetal bovine serum (FBS) (Thermo Scientific, Waltham, MA, United States) at 37°C in a humidified incubator with 5% CO_2_. For primary mouse Leydig cell culture, adult male CD-1 mice were euthanized, and the testes dissected and washed in phosphate-buffered saline (PBS). After removing connective tissue, the testes were enzymatically digested in 0.5% (*w*/*v*) collagenase IV (Sigma, St. Louis, MO, United States) for 25 min at 37°C. The reaction was stopped with FBS and the supernatant was filtered through a 400-mesh stainless steel filter. Cells were then collected and suspended in DMEM culture medium supplemented with 10% FBS at 37°C with 5% CO_2_. Testosterone levels were assayed using an ELISA kit (Jiancheng Bioengineering, Nanjing, China) according to the manufacturer’s instructions. PTC-209 and SB203580 were sourced from Selleck (Houston, TX, United States) and NAC was obtained from Sigma.

### Plasmids, siRNA, and Transfection

For the construction of plasmids overexpressing WT BMI1 and its nuclear localization signal (NLS2) mutant (BMI1-△NLS2), mouse *Bmi1* cDNA was PCR-amplified and then cloned into the pcDNA3.0 vector (Invitrogen, Carlsbad, CA, United States). For the construction of pGL6-*Map3k3* plasmids, both full length (−2000 ∼ + 100 relative to the transcriptional start site [TSS]; promoter-1) and partial (−1033 ∼ + 100, promoter-2 and −662 ∼ + 100, promoter-3) *Map3k3* promoter sequences were PCR-amplified from mouse genomic DNA and inserted into the pGL6 firefly luciferase reporter vector (Beyotime, Nantong, China). Both the BMI1-△NLS2 and *Map3k3* promoter mutant constructs were generated using the ClonExpress Ultra One Step Cloning Kit (Vazyme, Nanjing, China). *Bmi1* siRNA (5′-ACAAUGAAAGUUAAAAGUCGU-3′), *Map3k3* siRNA (5′-AGUCUAAUGCCUCUUGUUCAU-3′), and negative control (NC) siRNA (5′-ACGUGACACGUUCGGAGAA-3′) were synthesized by GenePharma (Shanghai, China).

The transient transfection of expression plasmids, luciferase reporter plasmids, or siRNAs was performed using Lipofectamine 3000 (Invitrogen) according to the manufacturer’s instructions. The transfected plasmids and siRNAs are indicated in the figures and figure legends. NC siRNA and empty vectors (EVs) were used as negative controls. After transfection, cells were utilized for measurements at the times indicated in the figure legends.

### Western Blot

Western blot was carried out as previously described (Zheng et al., 2014, 2015). Briefly, cell lysates were prepared using radioimmunoprecipitation assay (RIPA) buffer (Beyotime) containing protease inhibitors (Sigma). Protein was separated by SDS–PAGE and then transferred onto polyvinylidene difluoride membranes. After blocking, the membranes were incubated with the indicated primary antibodies (Supplementary Table 1) overnight at 4°C. The next day, the membranes were washed and then incubated with horseradish peroxidase (HRP)-conjugated secondary antibodies (Thermo Scientific) at room temperature (RT) for 2 h. Band signals were detected using an enhanced chemiluminescent substrate (Thermo Scientific) and subsequently analyzed by Image-Pro Plus Software.

### Cellular ROS Analysis

Cells were cultured in 96-well plates and treated as indicated in the figure legends. After washing three times with PBS, the cells were incubated with 20 M 2′,7′–dichlorofluorescein diacetate (DCFDA, Genmed Scientifics, Wilmington, DE, United States) for 40 min at 37°C. DCFDA is a fluorogenic dye that allows the measurement of hydroxyl, peroxyl, and other ROS activity within cells. Fluorescence intensity was measured on a fluorescence plate reader at the 488/525 nm excitation/emission wavelengths.

### Immunofluorescence

Cells were harvested, fixed in 4% (*w*/*v*) paraformaldehyde (PFA), blocked with 2% bovine serum albumin (BSA) (*w*/*v*) (Sigma) for 45 min at RT, and incubated with primary antibodies (Supplementary Table 1) overnight at 4°C according to a previously reported protocol (Zheng et al., 2018; Zhao et al., 2019). Next, the cells were rinsed with PBS and incubated with Alexa Fluor-conjugated secondary antibodies (Thermo Scientific) for 45 min. Nuclei were counterstained with DAPI (Beyotime). Images were acquired using a confocal laser microscope (Zeiss LSM800, Carl Zeiss, Oberkochen, Germany).

### RNA Extraction and Quantitative Real-Time Reverse Transcription-PCR

Cellular RNA was extracted using TRIzol reagent (Invitrogen) according to the manufacturer’s instructions. Total RNA was reverse transcribed into cDNA using a PrimeScript Reverse Transcription Kit (Vazyme) according to the instructions of the manufacturer. A total of 1 μL of the synthesized cDNA was mixed with a SYBR green mix (Vazyme) and primers in a final volume of 20 μL and applied to an ABI 7500 Real-Time PCR system (Applied Biosystems, Foster City, CA, United States). Relative gene expression levels were calculated using the 2^−ΔΔCt^ method with 18S rRNA serving as an internal control. The following primers were used: mouse *Map3k3*, forward 5′-GACTTCAGGACTCGCAGGC-3′ and reverse 5′-TGTTCATCCATGGTGGCGAT-3′; mouse 18sRNA, forward 5′-AAACGGCTACCACATCCAAG-3′ and reverse 5′-CCTCCAATGGATCCTCGTTA-3′.

### Luciferase Reporter Assay

Cells were cultured in 12-well plates and transiently transfected with 0.2 μg of the pGL6 luciferase reporter plasmid and *Bmi1* effector plasmid. Cells were also cotransfected with an internal control reporter plasmid containing *Renilla* luciferase (pRL-TK) to normalize the transfection efficiency. Two days after transfection, luciferase activity was detected using the Dual-Luciferase Reporter Gene Assay System (Beyotime).

### Chromatin Immunoprecipitation (ChIP)

The ChIP assay was performed using an EZ-ChIP Kit (Millipore, Billerica, MA, United States). Briefly, cells were harvested, washed, and cross-linked using 1% formaldehyde. The cells were then sheared into ∼500-bp fragments using a Branson Sonicator 250. The chromatin DNA–protein complex was reacted with the indicated antibodies (Supplementary Table 1). Approximately 10% of the starting material was used as the input. Immunoprecipitated DNA was analyzed by real-time PCR using the following primers: mouse *Map3k3* promoter, forward 5′-ATCGAGCCAAACCTTCCCTG-3′ and reverse 5′-CCACAACCCTCTGCTCAGTT-3′; and the mouse *Gapdh* gene, forward 5′-AACCCAAACTAACAGTTGTCCCAA-3′ and reverse 5′-ACTCCTTGGAGGCCATGTAGG-3′.

### Statistical Analysis

GraphPad Prism software was used for statistical analysis. Data obtained by the Student’s *t*-test or one-way ANOVA were presented as means ± standard deviation (SD) from at least three independent experiments. *p* < 0.05, *p* < 0.01, and *p* < 0.001 were set as the thresholds for statistical significance.

## Results

### BMI1 Positively Regulates Testosterone Production in MLTC-1 Cells

**FIGURE 1:**
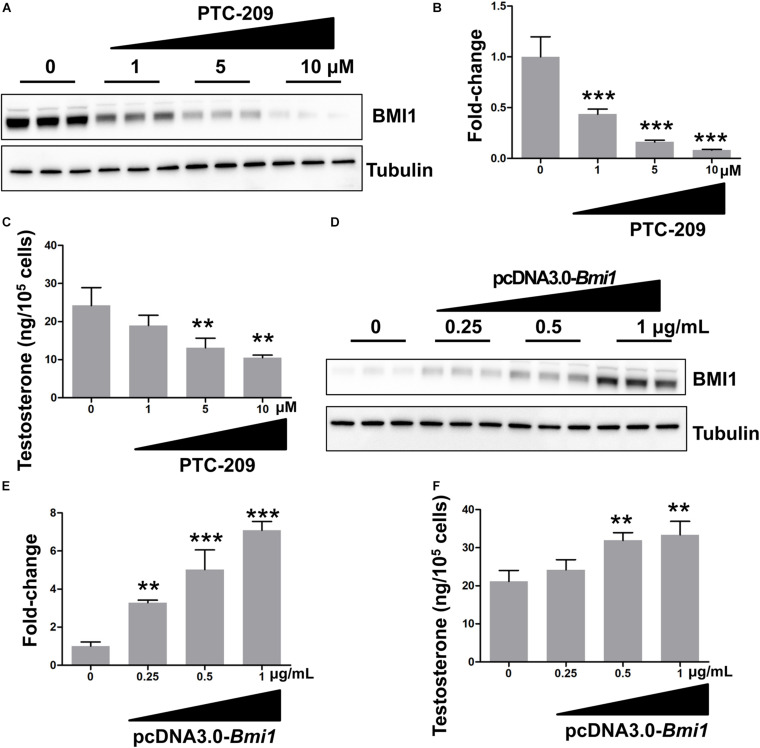
BMI1 promotes steroidogenesis in a dose-dependent manner. **(A)** MLTC-1 cells were treated with the indicated doses of PTC-209 for 48 h, followed by western blot analysis. **(B)** Quantification of **(A)**. **(C)** Assessment of testosterone levels in MLTC-1 cells treated with the indicated doses of PTC-209 after treatment with 1 IU/mL human chorionic gonadotropin (hCG) for 6 h. **(D)** MLTC-1 cells were transfected with the indicated concentrations of the pcDNA3.0-*Bmi1* plasmid for 48 h, followed by western blot analysis. **(E)** Quantification of **(D)**. **(F)** The assessment of testosterone levels in MLTC-1 cells transfected with the indicated concentrations of the pcDNA3.0-*Bmi1* plasmid after treatment with 1 IU/mL hCG for 6 h. **p < 0.01, ***p < 0.001.

In this study, we used the MLTC-1 mouse Leydig cell line for the analysis of the mechanistic and functional roles of BMI1 in steroidogenesis. This line stably and continuously produces androgenic hormones and is a commonly used model to study steroidogenesis (Abdou et al., 2014; Mendoza-Villarroel et al., 2014; Ayoub et al., 2016; Zhang et al., 2018). We first determined the effect of BMI1 on testosterone production by downregulating its expression using a BMI1-specific inhibitor, PTC-209 (Li et al., 2020; Zhu et al., 2020) or *Bmi1-*siRNA. We observed that both PTC-209 and *Bmi1*-siRNA treatment led to a substantial reduction in BMI1 levels (Figures 1A,B and Supplementary Figures 1A,B) and inhibited testosterone production in a dose-dependent manner (Figure 1C and Supplementary Figure 1C). In contrast, ectopic BMI1 expression significantly enhanced testosterone production (Figures 1D–F). These results strongly suggested that BMI1 is required for androgen synthesis.

### Both Cytoplasmic and Nuclear BMI1 Are Required for Steroidogenesis

**FIGURE 2:**
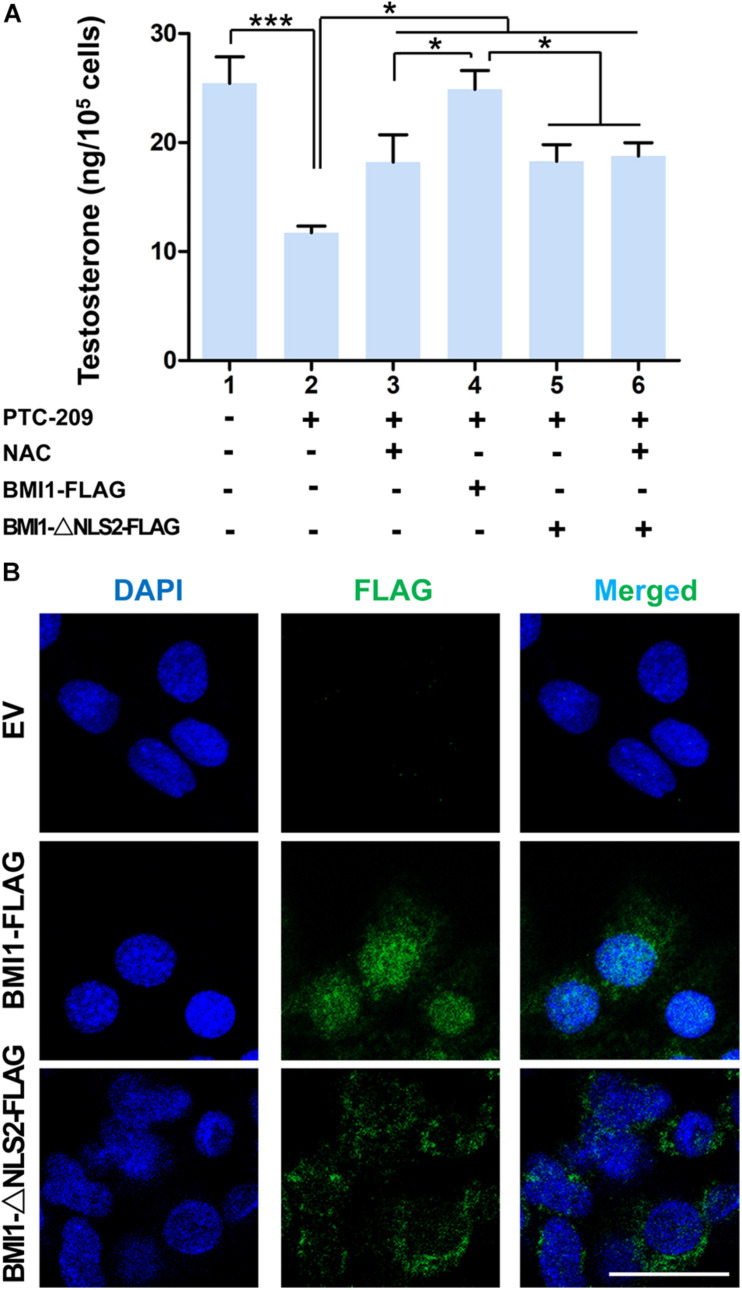
Both cytoplasmic and nuclear BMI1 are required for steroidogenesis. **(A)** The assessment of testosterone levels in MLTC-1 cells treated as indicated for 48 h. PTC-209, N-acetylcysteine (NAC), pcDNA3.0-*Bmi1-*flag, and pcDNA3.0-*Bmi1-△NLS2-*flag were used at the concentrations of 5 μM, 500 μM, 1 μg/mL, and 1 μg/mL, respectively. Testosterone levels were determined by ELISA after treatment with 1 IU/mL human chorionic gonadotropin (hCG) for 6 h. **(B)** Immunofluorescence staining for FLAG in MLTC-1 cells transfected with the pcDNA3.0-flag empty vector (EV), pcDNA3.0-*Bmi1-*flag, and pcDNA3.0-*Bmi1-△NLS2*-flag for 48 h. All constructs were used at the concentration of 1 μg/mL. Scale bar, 20 μm. *p < 0.05, ***p < 0.001.

In the cytoplasm, BMI1 inhibits ROS generation by regulating mitochondrial homeostasis (Banerjee Mustafi et al., 2016). In line with these observations, we found that BMI1-deficient MLTC-1 cells exhibited increased levels of oxidative stress and DNA double-strand breaks (DSBs) (Supplementary Figures 2A–C) (Gao et al., 2020). When PTC-209-treated MLTC-1 cells were further treated with the oxidant scavenger NAC (Liu et al., 2009; Chagraoui et al., 2011), testosterone production was significantly improved, which suggested that cytoplasmic BMI1 promoted steroidogenesis by maintaining redox homeostasis (Figure 2A). To further clarify the role of cytoplasmic BMI1 in steroidogenesis, we constructed a BMI1-△NLS2 plasmid (Figure 2B), which provided a powerful tool for investigating the role of BMI1 in the cytoplasm independently of its role in the nucleus (Cohen et al., 1996). We found that ectopic BMI1-△NLS2 expression in PTC-209-treated cells also enhanced testosterone production. Altogether, the above data revealed that cytoplasmic BMI1 has an important role in the promotion of steroidogenesis. However, when WT BMI1 was ectopically expressed in PTC-209-treated cells, the testosterone level was markedly higher than that in NAC- and/or BMI1-△NLS2-treated cells (Figure 2A). This indicates that nuclear-localized BMI1 also has a role in testosterone production.

### The Activation of p38 MAPK Is Independent of ROS in BMI1-Deficient Cells

**FIGURE 3:**
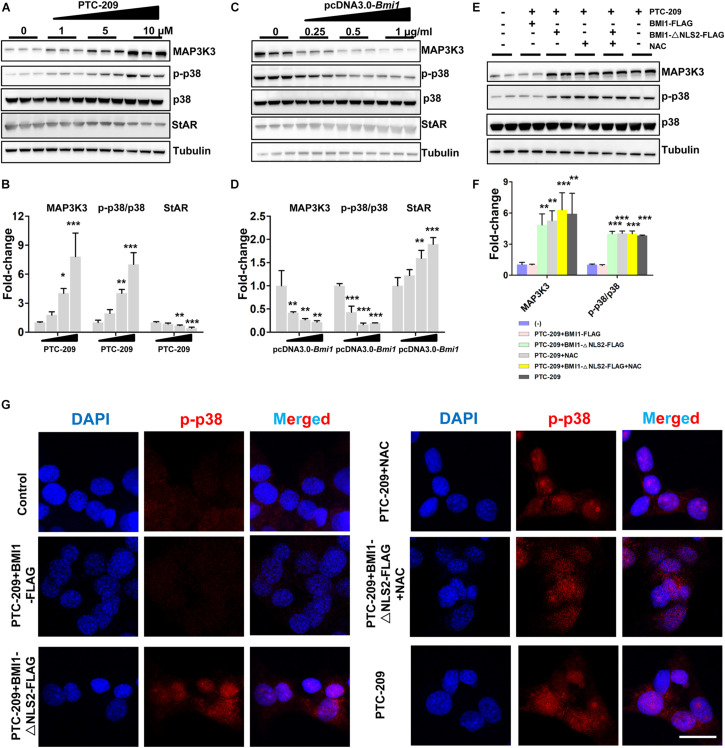
BMI1 inhibits the p38 MAPK pathway. **(A)** MLTC-1 cells were treated with the indicated concentrations of PTC-209 for 48 h, followed by western blot analysis of p38 MAPK expression. **(B)** Quantification of **(A)**. **(C)** MLTC-1 cells were transfected with the indicated concentrations of the pcDNA3.0-*Bmi1* plasmid for 48 h, followed by western blot analysis of p38 MAPK expression. **(D)** Quantification of **(C)**. **(E)** Western blot analysis of p38 MAPK expression in MLTC-1 cells treated as indicated for 48 h. PTC-209, pcDNA3.0-*Bmi1*-flag, pcDNA3.0-*Bmi1-△NLS2*-flag, and N-acetylcysteine (NAC) were used at the concentrations of 5 μM, 1 μg/mL, 1 μg/mL, and 500 μM, respectively. **(F)** Quantification of **(E)**. **(G)** Immunofluorescence staining for phospho-p38 in MLTC-1 cells treated as indicated for 48 h. PTC-209, pcDNA3.0-*Bmi1*-flag, pcDNA3.0-*Bmi1-△NLS2*-flag, and NAC were used at the concentrations of 5 μM, 1 μg/mL, 1 μg/mL, and 500 μM, respectively. DMSO combined with empty vector (EV) was used as the control. Scale bar, 20 μm. *p < 0.05, **p < 0.01, ***p < 0.001.

The p38 MAPK pathway is a negative regulator of steroidogenesis through the repression of *STAR* transcription (Zaidi et al., 2014). Here, we observed that the levels of p38 MAPK and its upstream regulator MAP3K3 were increased in PTC-209-treated MLTC-1 cells, whereas that of StAR was downregulated (Figures 3A,B). This indicated that the p38 MAPK pathway was activated. In contrast, ectopic BMI1 expression reduced the levels of both p38 and MAP3K3, while enhancing that of StAR (Figures 3C,D). However, the p38 MAPK pathway was not affected by the administration of NAC and/or BMI1-△NLS2 to PTC-209-treated cells (Figures 3E,F), whereas ROS production was markedly inhibited (Supplementary Figure 3). Only the ectopic expression of WT BMI1 could reduce p38 MAPK levels in PTC-209-treated cells (Figures 3E,F). To further analyze the expression of p38 MAPK in the differentially treated groups, we examined the expression of phosphorylated (activated) p38 (p-p38) in MLTC-1 cells using immunofluorescence (IF) (Figure 3G). The IF staining results were consistent with those obtained with western blotting (Figures 3E,F). Taken together, these data indicated that the p38 MAPK pathway is not activated by cytoplasmic BMI1-mediated oxidative stress in BMI1-deficient cells, suggesting that p38 MAPK is regulated by nuclear, and not cytoplasmic, BMI1.

### BMI1 Is Required for the Transcriptional Repression of Map3k3

**FIGURE 4:**
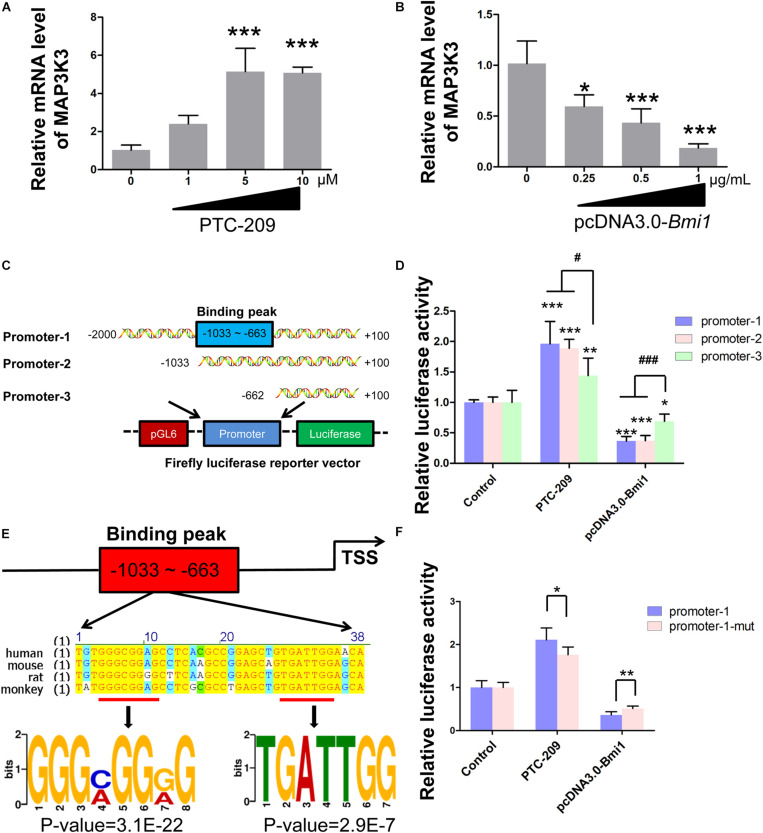
BMI1 inhibits *Map3k3* transcription. **(A)** MLTC-1 cells were treated with the indicated concentrations of PTC-209 for 48 h, followed by quantitative real-time reverse transcription-PCR (RT-qPCR) analysis of *Map3k3*. **(B)** MLTC-1 cells were transfected with the indicated concentrations of the pcDNA3.0-*Bmi1* plasmid for 48 h, followed by RT-qPCR analysis of *Map3k3*. **(C)** Schematic illustration of the firefly luciferase reporter constructs. **(D)** Luciferase activity assays in MLTC-1 cells transfected with luciferase reporter constructs and treated with PTC-209 (5 μM) or the pcDNA3.0-*Bmi1* (1 μg/mL) plasmid for 48 h. DMSO combined with empty vector (EV) was used as the control. *, compared with control; ^#^, compared with promoter-1 and -2. **(E)** Sequence analysis of the putative BMI1 binding region. **(F)** Luciferase activity assays in MLTC-1 cells transfected with luciferase reporter constructs containing promoter-1 and mutated promoter-1, PTC-209 (5 μM), or the pcDNA3.0-*Bmi1* (1 μg/mL) plasmid for 48 h. DMSO combined with empty vector (EV) was used as the control. *p < 0.05, **p < 0.01, ***p < 0.001; ^#^*p* < 0.05, ^###^*p* < 0.001.

In the nucleus, as a major member of polycomb repressive complex 1 (PRC1), BMI1 cooperates with E3 ubiquitin-protein ligase RING2 (RING1B) to monoubiquitinate histone H2A at K119, thereby mediating gene silencing (Wang et al., 2004; Morey et al., 2015). Therefore, we sought to determine whether BMI1 directly regulates the transcription of factors in the p38 MAPK pathway. We found that the mRNA expression of *Map3k3* was dose-dependently enhanced in PTC-209-treated MLTC-1 cells (Figure 4A), whereas the opposite was observed with ectopic BMI1 expression (Figure 4B). Additionally, bioinformatic analysis of publicly available BMI1 ChIP-seq data for the human MCF-7 cell line (ENCODE^1^, ENCSR966YYJ) revealed a BMI1-binding peak at the promoter region of *MAP3K3* (−359 ∼−742 bp upstream of the TSS) (Supplementary Figure 4), which corresponds to −633 ∼−1033 bp upstream of the TSS of the mouse *Map3k3* promoter (Supplementary Figure 5). Accordingly, we cloned both full-length (−2,000 ∼ + 100 bp [promoter-1]) and partial (−1033 ∼ + 100 [promoter-2] and −662 ∼ + 100 [promoter-3]) *Map3k3* promoter sequences into the pGL6 firefly luciferase reporter vector (Figure 4C). All three sequences could drive luciferase expression effectively; however, in the absence of the sequence encompassing the BMI1 binding peak (promoter-3), luciferase activity was significantly reduced compared with that for promoter-1 and promoter-2 (Supplementary Figure 6), suggesting that the sequence upstream of position −662 was vital for *Map3k3* transcription. Moreover, the inhibition of BMI1 markedly increased luciferase activity, but this effect was least pronounced with promoter-3 (Figure 4D); meanwhile, the overexpression of BMI1 elicited the opposite effect (Figure 4D). The above findings indicated that BMI1 mediates the transcriptional repression of *Map3k3* through binding to its core promoter region (−1033 ∼−663 upstream of the TSS). Surprisingly, the putative BMI1 binding sequence in mice showed only 59.7% similarity with that of humans (Supplementary Figure 5), suggesting that BMI1 could bind to the *Map3k3* promoter region through a specific motif. Further analysis of the region containing the BMI1 binding peak led to the identification of a 38-bp sequence that was highly conserved among humans, mice, rats, and monkeys (Figure 4E) and contained two functional motifs, namely, 5′-GGGMGGRG-3′ and 5′-TGATTGG-3′ (Figure 4E). To confirm this finding, we generated constructs containing the promoter-1 sequence that lacked the two motifs. A reporter assay revealed that the mutated promoter-1 significantly attenuated the BMI1-mediated transcriptional repression of *Map3k3* (Figure 4F). Collectively, these results demonstrated that BMI1 negatively regulates *Map3k3* transcription through binding to a set of specific motifs in its promoter region.

### Chromatin Remodeling by BMI1 Is Mediated Through Its Polycomb Function

**FIGURE 5:**
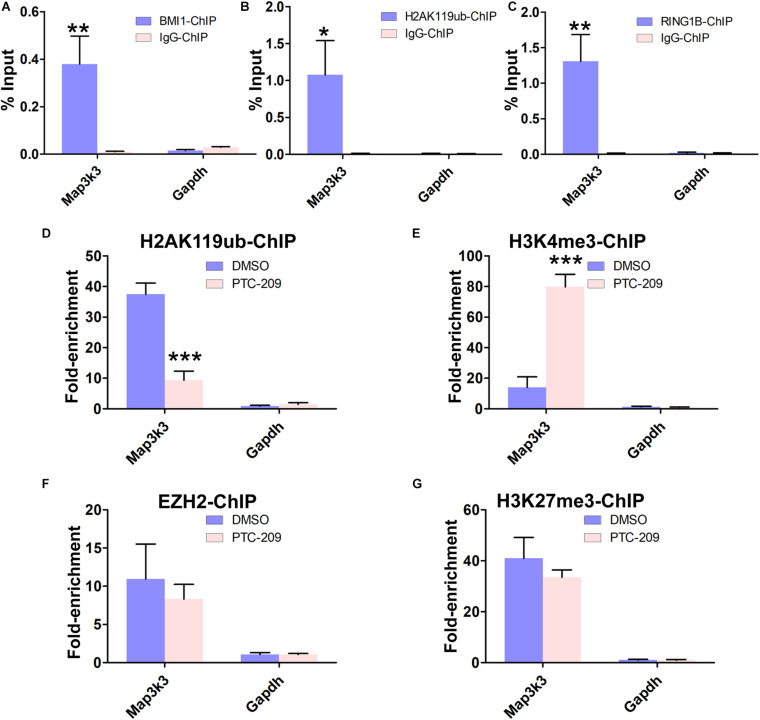
BMI1 inhibition alters the chromatin status at the BMI1 binding peak region in the *Map3k3* promoter. **(A–C)** ChIP-qPCR of BMI1-associated **(A)**, H2AK119ub-associated **(B),** and RING1B-associated **(C)** DNA sequences from the putative BMI1-binding region of the *Map3k3* promoter in MLTC-1 cells. The *Gapdh* gene was used as a negative control. **(D–G)** ChIP-qPCR of H2AK119ub-associated **(D)**, H3K4me3-associated **(E)**, EZH2-associated **(F)**, and H3K27me3-associated **(G)** DNA sequences in the putative BMI1-binding region of the *Map3k3* promoter in DMSO-treated or PTC-209-treated MLTC-1 cells. The *y*-axis represents enrichment relative to IgG controls. *p < 0.05, **p < 0.01, ***p < 0.001.

To obtain further insights into the mechanisms by which BMI1 mediates *Map3k3* transcriptional repression, we performed a ChIP assay in MLTC-1 cells using antibodies against BMI1, H2AK119ub, and RING1B. As expected, the BMI1 binding peak sequence was precipitated with BMI1, H2AK119ub, and RING1B (Figures 5A–C). In addition, after BMI1 depletion by PTC-209, significantly less H2AK119ub was precipitated with the region encompassing the BMI1 binding peak (Figure 5D), suggesting that the function of PRC1 was disrupted. Interestingly, bioinformatic analysis further revealed that the putative BMI1 binding region overlapped with the distribution of H3K4me3 (Supplementary Figure 7). In PTC-209-treated cells, the H3K4me3 level was measurably increased at the BMI1 binding peak region (Figure 5E). Taken together, these results demonstrated that BMI1 represses *Map3k3* expression through direct binding to its promoter region and modulating chromatin accessibility.

We next sought to determine whether the PRC2 complex can partly compensate for the disrupted PRC1 complex in PTC-209-treated cells. PRC2 is composed of the enhancer of zeste homolog 2 (EZH2), suppressor of zeste 12 (SUZ12), and embryonic ectoderm development (EED) proteins and contributes to the trimethylation of histone H3 at K27 (H3K27me3) (Sauvageau and Sauvageau, 2010). ChIP experiments indicated that PTC-209 treatment did not lead to any noticeable changes in EZH2 and H3K27me3 levels at the BMI1 binding peak-related region (Figures 5F,G).

### Knockdown of the P38 MAPK Pathway Restores Steroidogenesis in BMI1-Deficient MLTC-1 Cells

**FIGURE 6:**
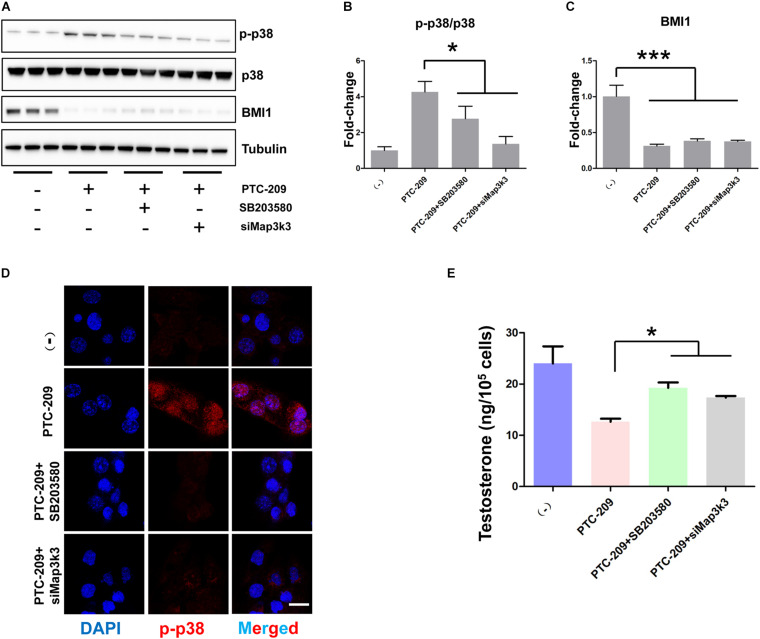
Inhibition of the p38 MAPK pathway rescued disrupted steroidogenesis. **(A)** Western blot analysis of phospho-p38 (p-p38) and BMI1 in MLTC-1 cells treated with PTC-209 (5 μM), SB203580 (10 μM), or siRNA targeting *Map3k3* (50 nM) for 48 h. **(B)** Quantification of **(A)**. **(C)** Quantification of **(A)**. **(D)** Immunofluorescence staining for p-p38 in MLTC-1 cells treated as indicated for 48 h. PTC-209, SB203580, and *Map3k3* siRNA were used at the concentrations of 5 μM, 10 μM, and 50 nM, respectively. **(E)** The assessment of testosterone levels in MLTC-1 cells treated as indicated for 48 h. PTC-209, SB203580, and *Map3k3* siRNA were used at the concentrations of 5 μM, 10 μM, and 50 nM, respectively. Testosterone levels were determined by ELISA after treatment with 1 IU/mL human chorionic gonadotropin (hCG) for 6 h. Scale bar, 20 μm. *p < 0.05.

To further test whether the p38 MAPK pathway contributes to the defective steroidogenesis observed in PTC-209-treated cells, we downregulated the p38 MAPK pathway by siRNA-mediated *Map3k3* silencing and the application of a widely used pharmacological inhibitor of p38, SB203580 (Li W. et al., 2017; He et al., 2020). Western blotting (Figures 6A–C) and IF (Figure 6D) analyses showed that both *Map3k3*-siRNA and SB203580 could efficiently inhibit p38 MAPK in PTC-209-treated cells. Meanwhile, western blot results also showed that SB203580 did not affect the levels of BMI1 (Figures 6A,C). Concomitantly, cells treated with PTC-209 and *Map3k3*-siRNA or SB203580 displayed significantly increased testosterone levels when compared with those in cells treated with PTC-209 alone (Figure 6E). The above data implied that excessively activated p38 signaling serves as a major repressor of BMI1-mediated steroidogenesis.

### BMI1 Epigenetically Orchestrates Steroidogenesis in Primary Mouse Leydig Cells

**FIGURE 7:**
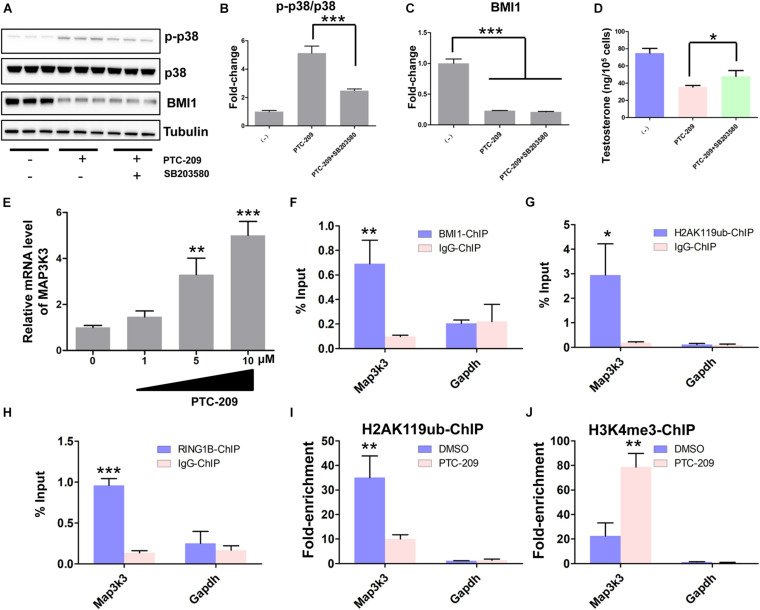
BMI1 epigenetically represses *Map3k3* in primary mouse Leydig cells. **(A)** Western blot analysis of phospho-p38 and BMI1 in Leydig cells treated with PTC-209 (5 μM) or SB203580 (10 μM) for 48 h. **(B)** Quantification of **(A)**. **(C)** Quantification of **(A)**. **(D)** The assessment of testosterone levels in Leydig cells treated with PTC-209 (5 μM) or SB203580 (10 μM) for 48 h. **(E)** Leydig cells were treated with the indicated concentrations of PTC-209 for 48 h, followed by quantitative real-time reverse transcription-PCR analysis of *Map3k3*. **(F–H)** ChIP-qPCR of BMI1-associated **(F)**, H2AK119ub-associated **(G)**, and RING1B-associated **(H)** DNA sequences in the putative BMI1-binding region of the *Map3k3* promoter in Leydig cells. The *Gapdh* gene was used as a negative control. **(I, J)** ChIP-qPCR of H2AK119ub- **(I)** and H3K4me3-associated **(J)** DNA sequences in the putative BMI1-binding region of the *Map3k3* promoter in DMSO-treated or PTC-209-treated Leydig cells. The *y*-axis represents enrichment relative to IgG controls. *p < 0.05, **p < 0.01, ***p < 0.001.

**FIGURE 8:**
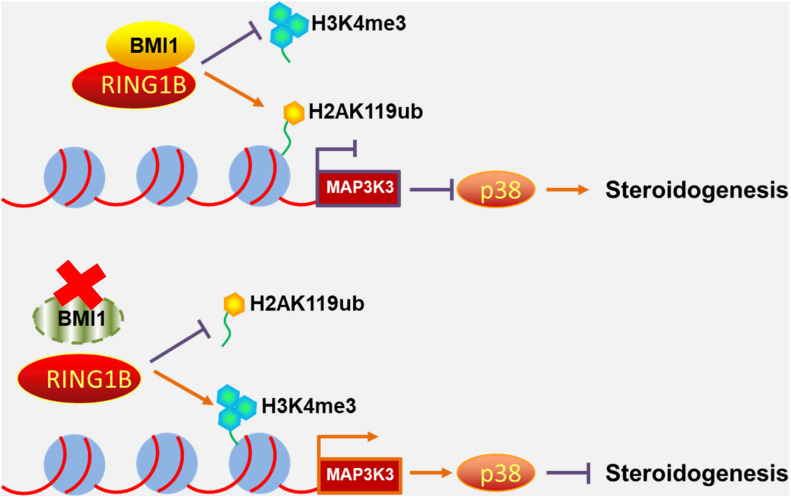
Schematic illustration of the working model for the role of BMI1 in steroidogenesis. In the nucleus, BMI1 is indispensable for PRC1 assembly. Together with RING1B, BMI1 facilitates the monoubiquitination of histone H2A at K119 to repress *Map3k3* expression, thereby promoting steroidogenesis. In the absence of BMI1, PRC1 function is disrupted and *Map3k3* is transcriptionally activated, thereby blocking steroidogenesis.

Primary mouse Leydig cells were used to further validate our findings in MLTC-1 cells. Consistent with the results obtained with MLTC-1 cells, when SB203580 was added to PTC-209-treated Leydig cells, p38 MAPK expression was inhibited, whereas the levels of testosterone were increased (Figures 7A–D); however, BMI1 levels were not affected (Figures 7A,C). Mechanistically, PTC-209 treatment does-dependently increased the mRNA expression levels of *Map3k3* in Leydig cells (Figure 7E). Additionally, a ChIP assay showed that BMI1, H2AK119ub, and RING1B were significantly enriched at the BMI1 binding peak region (Figures 7F–H), while the inhibition of BMI1 by PTC-209 markedly diminished the levels of H2AK119ub, while increasing those of H3K4me3 at the region containing the BMI1 binding peak in the *Map3k3* promoter (Figures 7I,J). Finally, we assessed the expression of p38 MAPK in testes from *Bmi1*-WT and *Bmi1*-KO mice. Compared with WT mice, the levels of MAP3K3 and p38 were upregulated in the testes of *Bmi1*-KO mice, whereas those of StAR were downregulated (Supplementary Figure 8). These results suggested that BMI1 also promotes steroidogenesis through the MAP3K3/p38 signaling pathway *in vivo*. Combined, our *in vitro* and *in vivo* findings demonstrated that BMI1 plays an essential role in promoting steroidogenesis by assembling PRC1, modulating chromatin accessibility, and repressing the expression of *Map3k3* (Figure 8).

## Discussion

Serum androgen levels decline in males as they age primarily as a result of functional alterations in the gonad. Aging can be defined as the accumulation of molecular damage in a cell or organism. ROS are known to be key sources of molecular damage and are a hallmark of aging (Lovell and Markesbery, 2007; Sena and Chandel, 2012; Wang and Hekimi, 2015; Kauppila et al., 2017). Several recent studies have implied that a relationship exists between BMI1 and ROS. For instance, it has been reported that BMI1 can prevent excessive ROS generation in neurons by repressing the pro-oxidant function of P53 (Chatoo et al., 2009). The results of a study by Liu et al. (2009) indicated that elevated intracellular ROS levels in *Bmi1*-KO mice were attributable to impaired mitochondrial function. More recently, Mustafi et al. reported that BMI1 exerts antioxidant activity in CP20 cells. The authors reported that mitochondria-localized BMI1 can stabilize mtRNA and enhance both electron transport chain function and ATP synthesis, and these functions are independent of its known functions in the nucleus (Banerjee Mustafi et al., 2016). Indeed, in this study, we observed that both the antioxidant NAC and cytoplasmic BMI1 could partly restore steroidogenesis in BMI1-deficient cells, which suggested that ROS is an important source of molecular damage during steroidogenesis. Besides these antioxidant functions of cytoplasmic BMI1, we also uncovered a novel epigenetic mechanism in steroidogenesis involving the BMI1-mediated silencing of the p38 MAPK pathway, which promoted steroidogenesis in both mouse MLTC-1 cells and primary Leydig cells. Mechanistically, BMI1 recruited the PRC1 complex to modify the chromatin structure surrounding the *Map3k3* promoter, thereby mediating the silencing of the *Map3k3* gene. Meanwhile, inhibition of the p38 MAPK pathway could improve steroidogenesis in PTC-209-treated MLTC-1 and Leydig cells. Altogether, our results have identified a role for the BMI1-MAP3K3-p38 MAPK axis in steroidogenesis, thereby providing potential therapeutic targets for the treatment of hypogonadism.

P38 MAPKs, one of the major MAPK subfamilies, are crucial signaling molecules that help transduce extracellular stimuli into cellular responses. The p38 MAPK pathway is activated by MAPK kinases (MAP2Ks or MKKs) such as MKK3 and MKK6; in turn, these MKKs are activated by MAPK kinase kinases (MAP3Ks or MEKKs), including apoptosis signal-regulating kinase 1 (ASK1), thousand-and-one amino acid kinase (TAO), leucine-zipper and sterile-alpha motif kinase (ZAK), transforming growth factor beta-activated kinase 1 (TAK1), and MAP3K3 (Wagner and Nebreda, 2009; Arthur and Ley, 2013). The p38 MAPK pathway is known to act as a ROS sensor. Stimuli such as pathogen attack, ultraviolet (UV) radiation, wounding, and chemotherapeutic agents can induce ROS generation, thereby triggering p38 MAPK pathway activation, which further leads to the modulation of cellular growth and metabolism (Finkel and Holbrook, 2000; Patel et al., 2019). Studies have revealed that ROS-mediated activation of the p38 MAPK pathway impairs steroidogenesis through the transcriptional repression of the *STAR* gene and the feedback inhibition of a key steroidogenic transcription factor, CREB (Zaidi et al., 2014; Li J. et al., 2017). Besides ROS, substantial evidence has indicated that follicle-stimulating hormone (FSH) can also induce the phosphorylation and activation of p38 MAPK. Moreover, the inhibition of p38 MAPK activity using the p38 MAPK inhibitor SB203580 enhances FSH-induced steroidogenesis in rat granulosa cells (Yu et al., 2005). Intriguingly, in our study, PTC-209-induced ROS production in MLTC-1 cells did not contribute to the activation of the MAP3K3-p38 axis, as evidenced by the fact that treatment with the oxidant scavenger NAC and/or the ectopic expression of BMI1-△NLS2 failed to alleviate the activation of p38 MAPKs. This suggested that MAP3K3 was not an oxidative stress sensor in this situation. Indeed, specific MAP3Ks are sometimes associated with specific stresses. For instance, TAK1 activation is always cytokine receptor-dependent (Sorrentino et al., 2008; Yamashita et al., 2008). Moreover, MAP3K1 mediates p38 activation following UV or peptidoglycan stimulation (Zhuang et al., 2006), while ASK1 activates p38 under conditions of oxidative stress (Dolado et al., 2007).

The identification of histone “codes” has greatly contributed to our understanding of gene regulation networks in almost all stages of development (Jambhekar et al., 2019). Histone modifications must be coordinately regulated for accurate cell fate determination, and dysregulated histone modifications always lead to aging, cancer, or other diseases (Booth and Brunet, 2016; Husmann and Gozani, 2019; Michalak et al., 2019). One histone modification can be repressed or enhanced in the presence or absence of another. For instance, LSD1-mediated demethylation of histone H3K4 is activated upon the removal of histone acetylation (Shi et al., 2005; Lee et al., 2006). Additionally, the transcriptional regulator FOXP3 promotes both H3K4 trimethylation and H4K16 acetylation through displacing the demethylase PLU1 and recruiting the acetyltransferase MOF (Katoh et al., 2011). In the present study, BMI1 knockdown led to a significant decrease in H2AK119ub and an increase in H3K4me3 levels at the promoter region of *Map3k3*, which derepressed its expression. In mouse embryonic fibroblasts (MEFs), BMI1 deficiency led to the removal of H2AK119ub and the enrichment of H3K4me3 at BMI1 binding regions, which was accompanied by the upregulation of the expression of the corresponding genes. In contrast, gene expressions were not affected when there was a decrease in H2AK119ub but no change in H3K4me3 levels at BMI1 binding regions in BMI1-deficent MEFs (Zhou et al., 2016). Collectively, these findings suggested that H3K4me3 contributes to the derepression of BIM1-regulated genes.

## Conclusion

In conclusion, to the best of our knowledge, this study represents the first loss- and gain-of-function analysis of the role of BMI1 in steroidogenesis. Our study uncovered a novel epigenetic mechanism in steroidogenesis involving BMI1-mediated gene silencing and provides potential therapeutic targets for the treatment of hypogonadism.

## Data Availability Statement

The original contributions presented in the study are included in the article/Supplementary Material, further inquiries can be directed to the corresponding author/s.

## Ethics Statement

The animal study was reviewed and approved by The Ethics Committee of Nanjing Medical University.

## Author Contributions

YW and HL performed most of the experiments. HZ, CS, TG, MLin, XD, and JO performed some of the experiments. HL and MLiu analyzed the data. BZ, XH, FS and JY initiated the project and designed the experiments. JY and BZ wrote the manuscript. All authors read and approved the final manuscript.

## Conflict of Interest

The authors declare that the research was conducted in the absence of any commercial or financial relationships that could be construed as a potential conflict of interest.

## Footnotes

1 www.encodeproject.org
