# Supplementary material for: BMI1 Drives Steroidogenesis Through Epigenetically Repressing the p38 MAPK Pathway
Source: Front Cell Dev Biol. 2021 Apr 13;9:665089. doi: 10.3389/fcell.2021.665089 (PMC8076678; doi:10.3389/fcell.2021.665089)
Supplement: Supplementary file 1 [file Data_Sheet_1.docx]

Supplementary Material

# Supplementary Figures


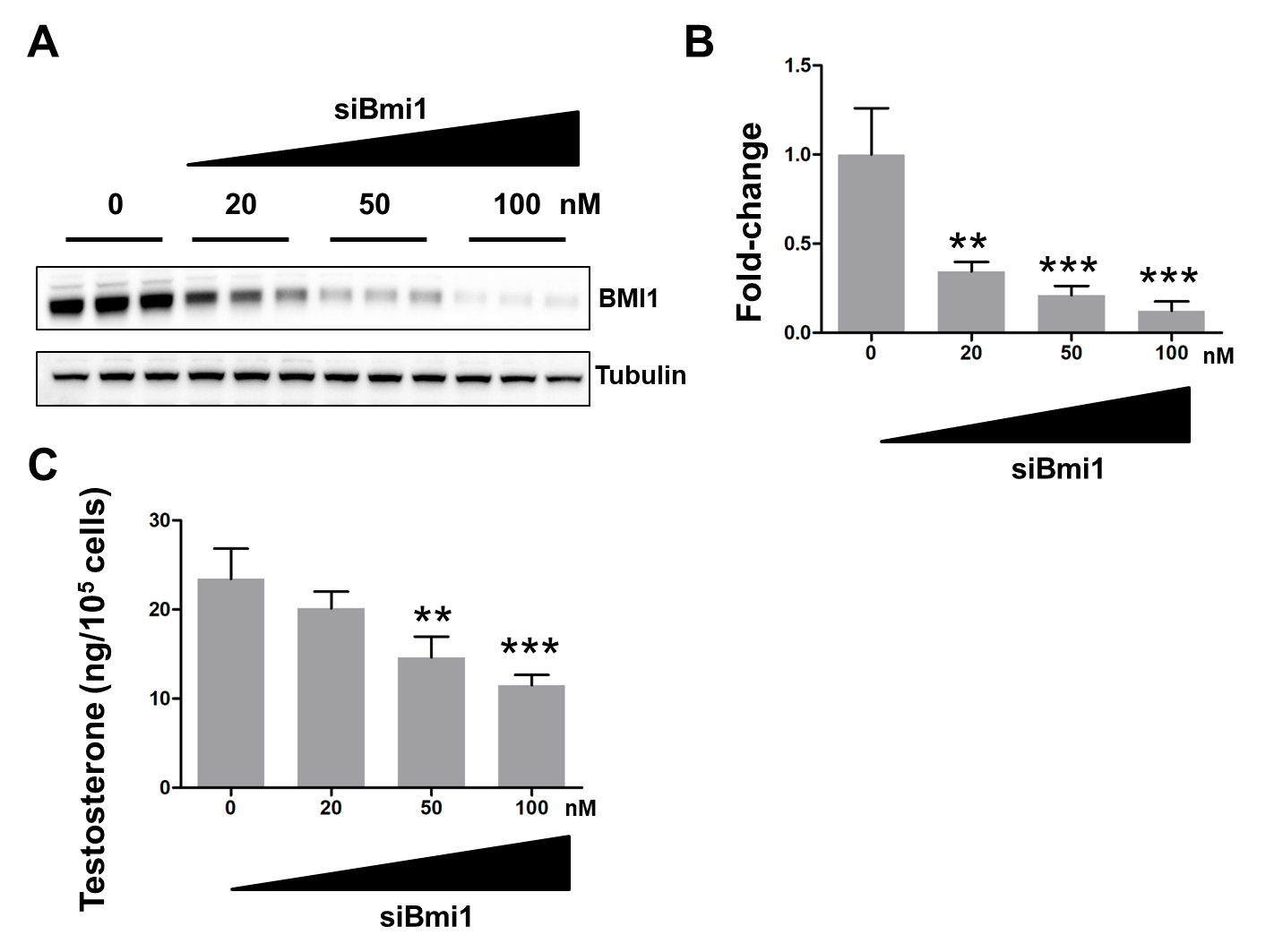


**Supplementary Figure 1**. siRNA-mediated *Bmi1* silencing. **(A)** MLTC-1 cells were treated with the indicated concentrations of *Bmi1* siRNA (siBmi1) for 48 h, followed by western blot analysis. **(B)** Quantification of **(A)**. **(C)** The assessment of testosterone levels in MLTC-1 cells treated with the indicated concentrations of siBmi1 after treatment with 1 IU/mL human chorionic gonadotropin (hCG) for 6 h. ***p* < 0.01, ****p* < 0.001.

**
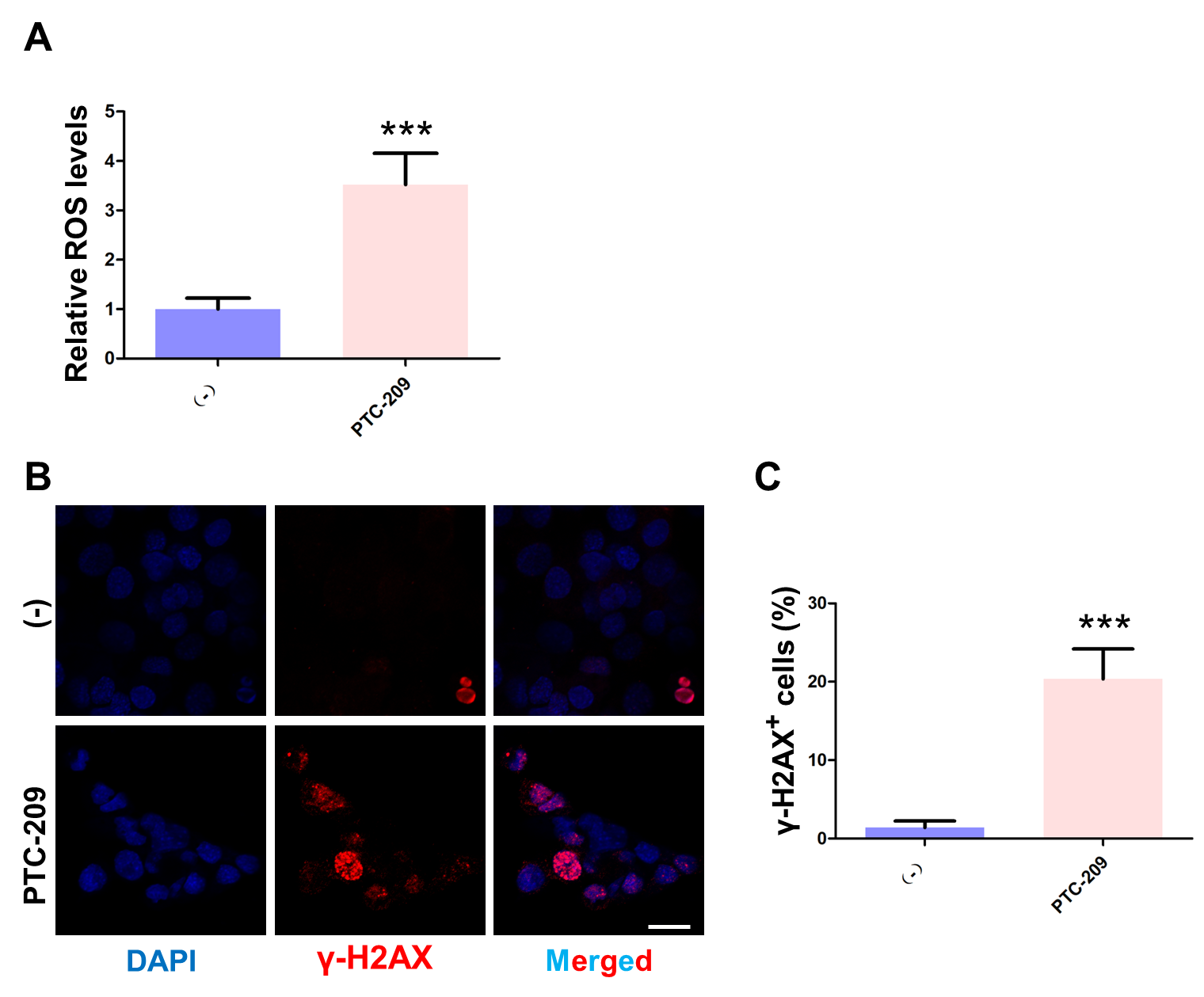
**

**Supplementary Figure 2**. BMI1 deficiency increases reactive oxygen species (ROS) production and the number of double-strand breaks (DSBs). **(A)** The assessment of ROS levels in MLTC-1 cells treated with PTC-209 (5 μM) or DMSO (−) for 48 h. **(B)** Immunofluorescence staining for γ-H2AX in MLTC-1 cells treated with PTC-209 (5 μM) or DMSO (−) for 48 h. **(C)** Quantification of **(B)**. ****p* < 0.001.

**
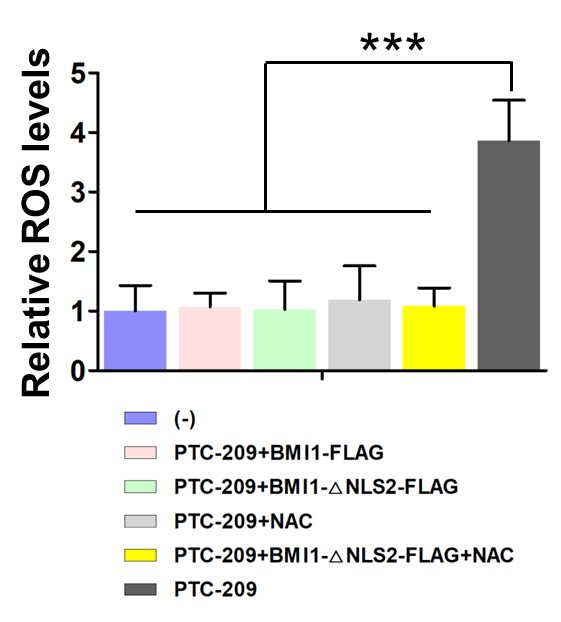
**

**Supplementary Figure 3**. The assessment of reactive oxygen species (ROS) levels in MLTC-1 cells treated as indicated for 48 h. PTC-209, pcDNA3.0-*Bmi1*-flag, pcDNA3.0-*Bmi1-△NLS2*-flag, and N-acetylcysteine (NAC) were used at the concentrations of 5 μM, 1 μg/mL, 1 μg/mL, and 500 μM, respectively. ****p* < 0.001.

**
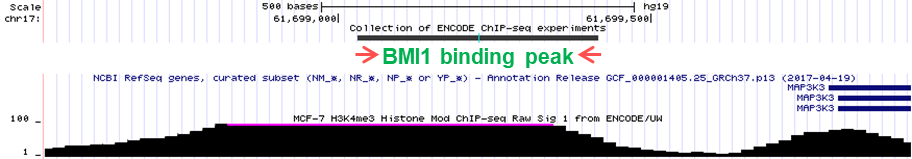
**

**Supplementary Figure 4**. The distribution of human H3K4me3 and BMI1-binding regions at the *MAP3K3* locus. All chip-seq data were obtained from the ENCODE database and were visualized using the UCSC genome browser (http://genome.ucsc.edu). *MAP3K3* gene structure was annotated based on known genes in the UCSC database and NCBI RefSeq genes. H3K4me3 indicated promoter regions.


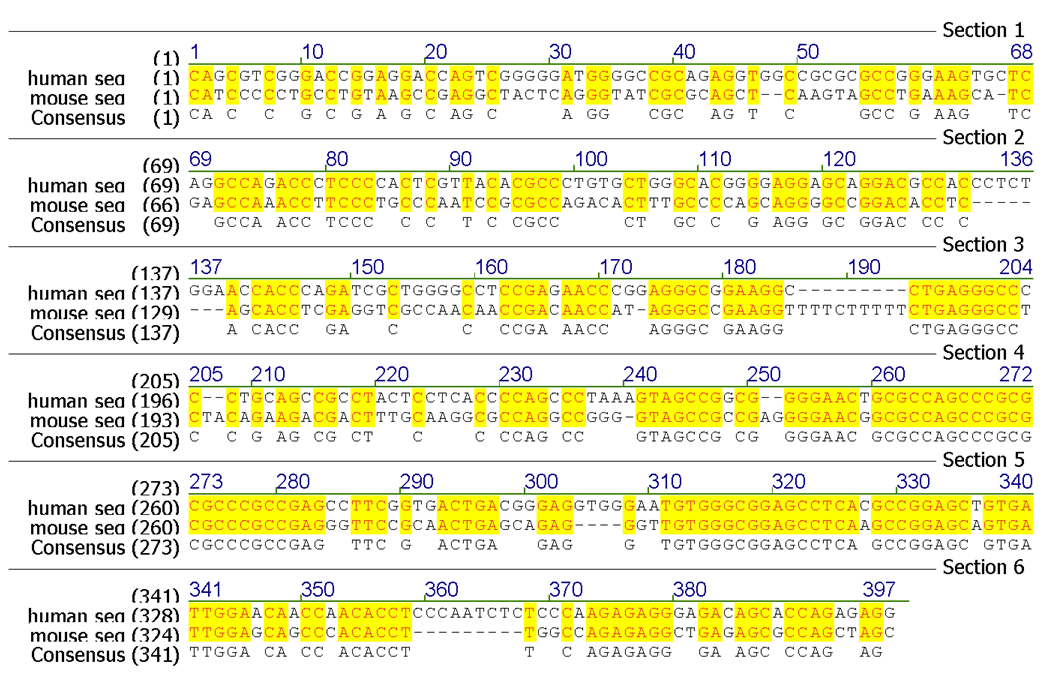


**Supplementary Figure 5**. Sequence alignment of human and mouse regions associated with the BMI1 binding peak in the human *MAP3K3* promoter. The human product is 59.7% identical to that of the mouse.

**
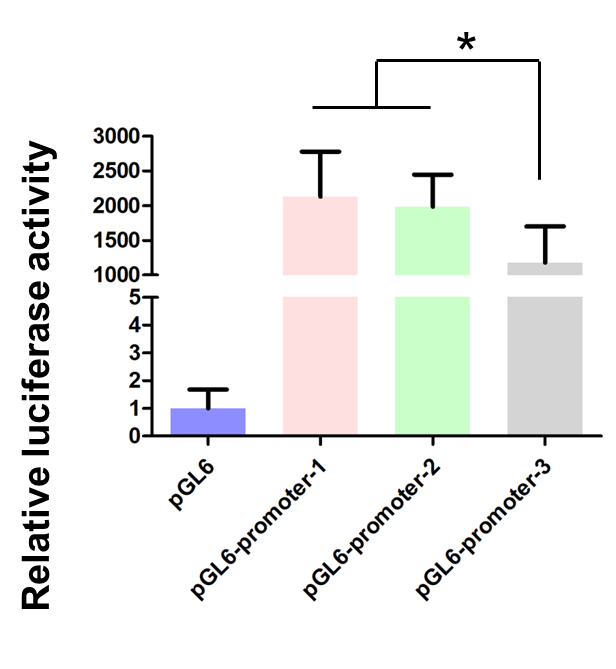
**

**Supplementary Figure 6**. Luciferase activity assays in MLTC-1 cells. MLTC-1 cells were transfected with pGL6 EV (empty vector), pGL6-promoter-1, pGL6-promoter-2, or pGL6-promoter-3 constructs for 48 h. **p* < 0.05.

**
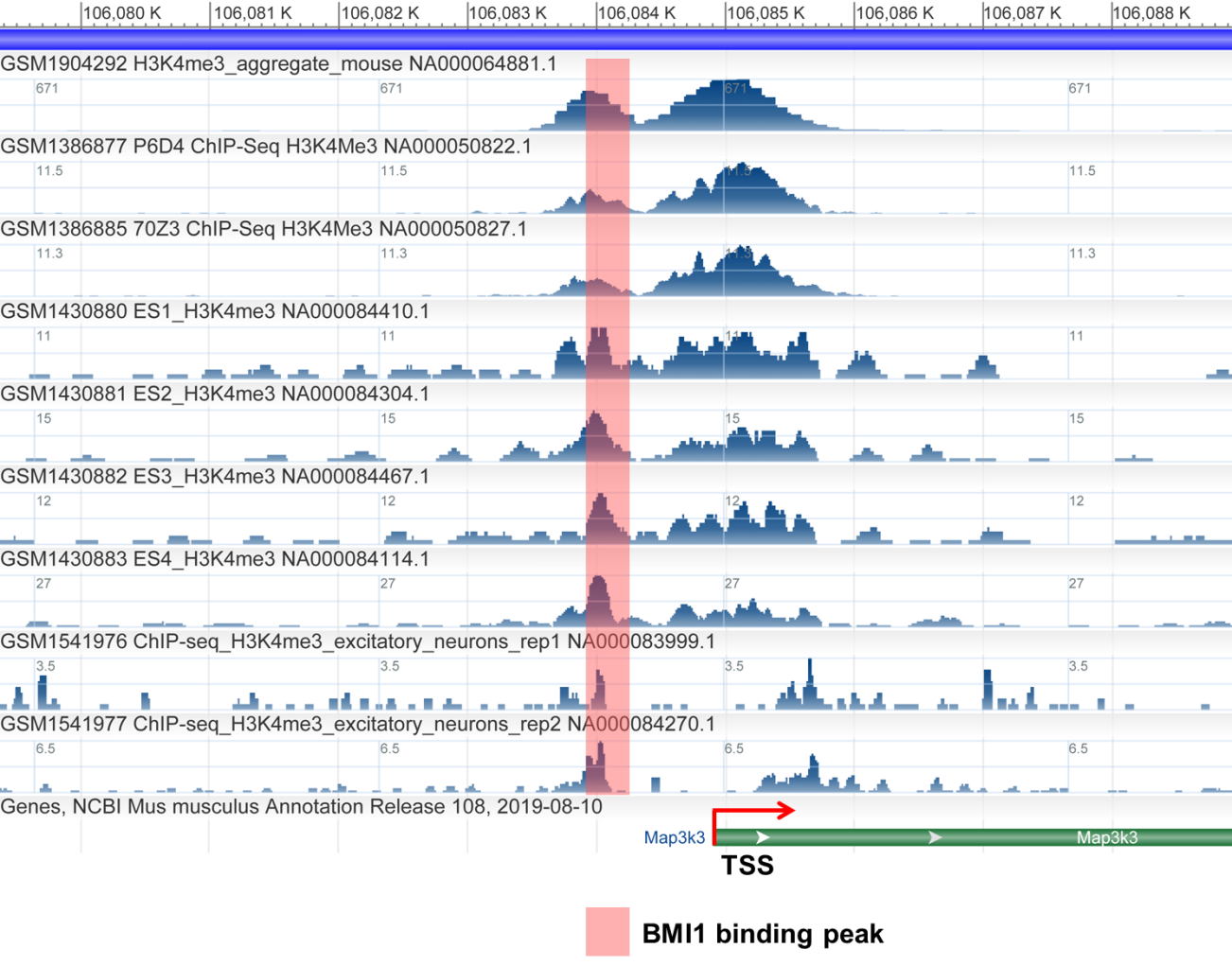
**

**Supplementary Figure 7**. The distribution of mouse H3K4me3 at the putative BMI1 binding region in the *Map3k3* promoter. ChIP-seq data were acquired and visualized at NCBI (<https://www.ncbi.nlm.nih.gov/gene/>).


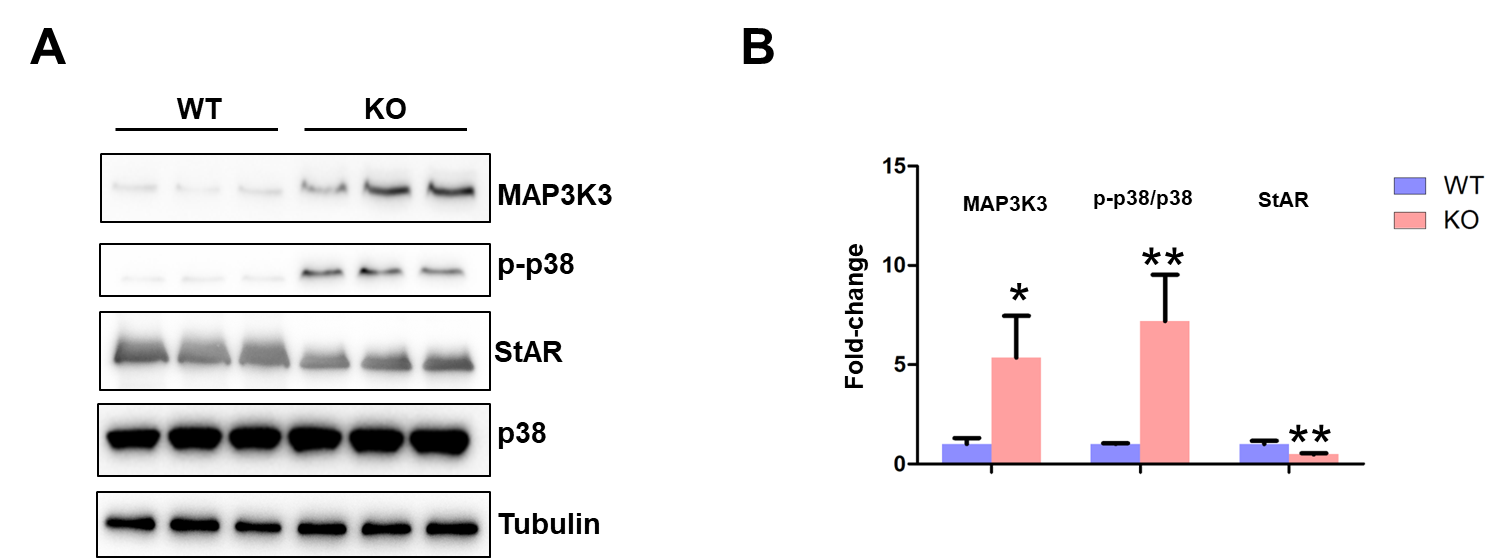


**Supplementary Figure 8**. p38 MAPK signaling is activated in the testes of *Bmi1*-knockout (KO) mice. **(A)** Western blot analysis of p38 MAPK expression in adult testes of *Bmi1*-wild-type or *Bmi1-*KO mice. **(B)** Quantification of **(A)**. ****p* < 0.001.

# Supplementary Table

**Supplementary Table 1. Antibodies information**

| Antigen | Source | Company | Application | Dilution |
| --- | --- | --- | --- | --- |
| BMI1 | Mouse | Milipore | WB; ChIP | 1:1000; 10μg/ChIP |
| FLAG | Mouse | Sigma | IF | 1:1000 |
| Tubulin | Mouse | Beytime | WB | 1:10000 |
| ub-Histone H2A(K119) | Rabbit | Cell Signal | ChIP | 10μg/ChIP |
| MAP3K3 | Rabbit | Cell Signal | WB | 1:1000 |
| p-p38 | Rabbit | Cell Signal | WB;IF | 1:1000;1:500 |
| p38 | Rabbit | Cell Signal | WB | 1:1000 |
| StAR | Rabbit | Cell Signal | WB | 1:1000 |
| RING1B | Mouse | Active Motif | ChIP | 5μg/ChIP |
| H3K4me3 | Rabbit | Milipore | ChIP | 5μg/ChIP |
| EZH2 | Mouse | BD | ChIP | 5μg/ChIP |
| H3K27me3 | Rabbit | Milipore | ChIP | 5μg/ChIP |
| γ-H2AX | Mouse | Abcam | IF | 1:1000 |
|  |  |  |  |  |
